# A Highly Secure IoT Firmware Update Mechanism Using Blockchain

**DOI:** 10.3390/s22020530

**Published:** 2022-01-11

**Authors:** Woei-Jiunn Tsaur, Jen-Chun Chang, Chin-Ling Chen

**Affiliations:** 1 Computer Center, National Taipei University, New Taipei City 237303, Taiwan; 2 Department of Computer Science and Information Engineering, National Taipei University, New Taipei City 237303, Taiwan; jcchang@mail.ntpu.edu.tw; 3 School of Computer and Information Engineering, Xiamen University of Technology, Xiamen 361005, China; 4 School of Information Engineering, Changchun Sci-Tech University, Changchun 130600, China; 5 Department of Computer Science and Information Engineering, Chaoyang University of Technology, Taichung City 413310, Taiwan

**Keywords:** Internet of Things (IoT), blockchain, smart contract, information security

## Abstract

Internet of Things (IoT) device security is one of the crucial topics in the field of information security. IoT devices are often protected securely through firmware update. Traditional update methods have their shortcomings, such as bandwidth limitation and being attackers’ easy targets. Although many scholars proposed a variety of methods that are based on the blockchain technology to update the firmware, there are still demerits existing in their schemes, including large storage space and centralized stored firmware. In summary, this research proposes a highly secure and efficient protection mechanism that is based on the blockchain technology to improve the above disadvantages. Therefore, this study can reduce the need of storage space and improve system security. The proposed system has good performance in some events, including firmware integrity, security of IoT device connection, system security, and device anonymity. Furthermore, we confirm the high security and practical feasibility of the proposed system by comparing with the existing methods.

## 1. Introduction

With the popularity of Internet of Things (IoT) devices, people’s lives are gradually inseparable from IoT devices. However, the increasing popularity of IoT devices has been accompanied by several challenges, including security and update service availability. In terms of security, IoT devices are mostly less secure than personal computers with many protection mechanisms, so they often become the target of hackers [1]. Recently, several serious IoT device attacks have occurred, such as many IoT devices that were controlled by hackers to launch attacks of distributed denial of service (DDoS) [2,3]. Moreover, due to the attack of unsafe ports, the company FCA US LLC (Fiat Chrysler Automobiles United States Limited Liability Company) urgently recalled products that were equipped with car networking systems [4]. On the other hand, hackers invaded medical devices to obtain patient personal data [5]. Therefore, the above events repeatedly reminded us of the importance of IoT device security.

According to “Strategy Analytics”, the number of IoT devices will exceed 50 billion after 2020 [6]. A huge number of IoT devices bring convenience to life, but users often forget the importance of information security, such as using default passwords, not regularly updating firmware, etc. Using IP camera as an example, hackers can see many monitor screens using the default password on the network [7], including many corporate offices and commercial warehouses on the screen in the world. As a result, the protection of IoT device security is a major issue.

Traditionally, a manufacturer provides IoT devices with firmware update files, but it has the limitations of manufacturer’s server size and bandwidth. As the number of IoT devices has been increasing, manufacturers cannot update the device quickly and instantly, and thus they make many devices exposed to danger. IoT devices are widely used, and most of them are lightweight devices and lack a strong antivirus mechanism. When devices are invaded by hackers, they will often reinstall the firmware to ensure that the devices can operate normally [8]. With the advancement of technology, to defend against endless attacks, a firmware update is an extremely crucial solution. Nevertheless, there are still several shortcomings about the firmware update of IoT devices. It is hard to confirm that the manufacturer deploys the firmware. It will be harmful to a device if the device obtains the wrong firmware. Consequently, a secure IoT firmware update is concerned by IoT research community. Using peer-to-peer system solves the bandwidth limitation, but it also brings some doubts about security. Because communication protocol has some vulnerabilities [9], it also increases the risk while transmitting firmware update files. While the process for updating IoT devices is vital to ensure security aspects, the requirements of IoT scenarios make it difficult to develop a comprehensive solution. The following challenges need to be tackled for a secure firmware update process:

(1) Integrity: The firmware update process must ensure the integrity of a firmware update. When an adversary intercepts the firmware update file transmission and replaces it with malicious malware, the device will install the malware instead of the original update file.
(2) Downloading firmware update: Most of the current proposals to download a firmware update are based on a typical centralized client-server architecture. These proposals commonly use the over-the-air (OTA) update for IoT devices because it is quick and convenient for users. They can connect the device to a computer and download the updates through the Internet. However, since most OTA updates adopt a centralized way, they are vulnerable to a single point of failure and generate the latency issue. Server failure and network latency will cause the delay of critical update. Therefore, considering the scalability and heterogeneity of IoT devices, the use of decentralized models can assist in this process to minimize the overall network overhead and eliminate the single point of failure.
(3) Request for firmware update: When massive IoT devices request firmware updates at the same time, how to cope with the update service availability of massive IoT devices is an essential issue.
(4) Authentic firmware: The firmware update file needs to be checked to make sure it is not malicious.

The authentication, non-repudiation, integrity, and availability are the four characteristics in information security. In order to create a highly secure system, all major companies have their own security protection mechanisms. Taking the IoT device manufacturer as an example, the manufacturer will store the updated firmware file in the file server, which has great protection to ensure the security of the file [10]. By using the blockchain technology, it can reduce the burden on the file server, and it can also keep information of the system secure. Therefore, there is some research about blockchain-based firmware file transmission systems [11,12,13]. By combining the technology of blockchain, the advantages of blockchain are used in file updating applications, and it can make the system more available and secure. Although many scholars [11,12,13] have proposed the method of blockchain technology to improve the demerits of traditional methods, there are still potential risks and deficiencies. Lee and Lee [11] proposed a blockchain-based system for updating firmware. However, in their method, IoT devices are the blockchain nodes, so the device stores too many files. Since IoT devices are resource-constrained, it is difficult for Lee and Lee’s scheme [11] to be achieved in a real IoT environment. Boudguiga et al. [12] combined antivirus companies as antivirus nodes with the blockchain system for checking files to ensure that the files on the blockchain are not installed with malware. And each antivirus nodes also store the firmware. However, Boudguiga et al.’s method [12] has large storage problem, because it must save all firmware in each blockchain node to maintain the integrity, which will lead to the need of large storage. In addition, whenever firmware files are updated, they must be sent to a few antivirus nodes for inspection. In such a way, antivirus nodes have been overburdened. On the other hand, Yohan and Lo [13] used the smart contract in Ethereum to keep an autonomous firmware update, and it improved Boudguiga et al.’s method [12] by storing the uniform resource identifier instead of firmware in each node. The IoT devices fetch firmware through the uniform resource identifier, which can reduce the needs of storage space in each node. The above practices improve the storage but sacrifice security. Because the uniform resource identifier is not equal to the firmware, it is unable to guarantee the security of the firmware even if the uniform resource identifier is difficult to be tampered.

Since the current firmware update methods for IoT devices still have shortcomings, in this study we combine the blockchain technology with a decentralized database to improve the deficiencies of existing methods. The proposed approach is based on the blockchain technology to ensure the security of the firmware, including source verification, firmware integrity, and traceability. Furthermore, the proposed approach can reduce the storage space requirements, prevent firmware tampered after it is deployed, and lessen the burden of the firmware manufacturers’ servers.

The rest of this paper is organized as follows. In Section 2, we first introduce the existing methods of IoT firmware attacks, and we then discuss the well-known blockchain development platform. Section 3 illustrates the system architecture and mechanism proposed in this study. In Section 4, security analyses are conducted for the proposed system and compared with other previous methods. In Section 5, we show the experimental evaluation and comparison with other methods. The final section will list the contributions made in this study.

## 2. Related Work

In this section, we first summarize the relevant IoT firmware attack to help understand the vulnerabilities and risks of IoT devices, and we further survey the blockchain technology to propose a potential solution for secure IoT firmware update.

### 2.1. IoT Firmware Attack

In order to improve the security of the IoT device, it is necessary to understand the vulnerabilities and risks of IoT devices. The following describes the critical attack methods.

#### 2.1.1. Default Password Attack

The surveillance cameras are one of the most common IoT devices in our lives. However, many people do not change camera default passwords so that Mirai, a kind of virus, can control the devices. In 2016, a hacker attacked the website, “Krebs on Security,” by using Mirai-infected IoT devices and successfully hacked its servers. Later, many well-known websites were hacked, including GitHub, Twitter, and Reddit. The DDoS derived from Mirai has reached the scale of 1 Tbps and has a worldwide influence [14]. The author published the source code of Mirai after the attack [2], which caused many scholars in the field of information security to study Mirai [15,16].

Mirai used the built-in dictionary file to try to gain control of the IoT device. If the device is successfully compromised, it will be implanted with malicious programs, and the infected device will still operate normally. However, its bandwidth consumption will become larger due to Mirai-infected devices that still function, so many devices become part of the botnet without their knowledge.

#### 2.1.2. Vehicle Network Attack

The company FCA US LLC, a major car manufacturer, developed the “Uconnect System” to launch a networked product. However, after hackers invaded the system, they found that the system used some unsafe ports, allowing the hacker to operate the car door, wiper operation, and brake system. Therefore, manufacturers recalled the product for maintenance [4]. The development of technology and technology itself will bring convenience to life, but at the same time there will be related vulnerabilities. Car networking is one of the famous examples. The importance of a firmware update is further highlighted by the changes in the environment.

#### 2.1.3. Printer Attack

The printer is one of the tools used in life, but there were incidents of being controlled by hackers and printing threatening letters. Since most connected printers use an external physical IP, their information is exposed on the network so that an attacker can manipulate it through the port used by the printer [17]. In order to prove that there are many loopholes in the networked device, a white-handed hacker “Stackoverflowin” successfully used the printer to invade 160,000 connected printers, and at the same time, the invaded printer printed the robot’s pattern. It reminds users that the device is vulnerable and should be processed as soon as possible.

### 2.2. Blockchain Technology

Blockchain technology that is applied to the cryptocurrency is called Bitcoin [18,19] at first. Cryptocurrency is different from currency because cryptocurrency does not have a central bank to decide the value. With the rise of Bitcoin, people have seen the technology of blockchain, and major companies are scrambling to develop the relevant applications of the blockchain. There are many blockchain development platforms, such as Ethereum and Hyperledger, and each of them have different characteristics. Ethereum is a blockchain platform developed by Vitalik Buterin. The cryptocurrency on Ethereum is Ether. The biggest difference between Ethereum and Bitcoin is a smart contract that allows Ethereum to be able to deploy applications on blockchain. According to different smart contracts, Ethereum can perform many applications, such as playing games. Therefore, Ethereum has another name, “blockchain 2.0.” Ethereum has led the application of blockchain technology to a new generation. In the case of the remaining characteristics of blockchain, Ethereum extends to general applications, which is called “Decentralized Applications”. Ethereum is relatively early among a variety of platforms, and its stability and popularity are quite high [20,21,22,23]. On the other hand, Hyperledger is a blockchain interoperability application promoted by the Linux Foundation. It has many platforms, such as Fabric [24] led by IBM. The system takes into account the structure of the enterprise and makes different protocol based on different network architectures, such as “Byzantine Fault Tolerance (BFT),” to achieve privacy protection. Because it is more private, it usually makes application in the financial industry that is sensitive to personal privacy [25].

IOTA is a blockchain platform developed by Dominik Schiener’s team [26]. It is mainly used on the Internet of Things, providing payment and file storage functions. The underlying layer uses decentralized ledger technology, called Tangle, to make transactions [27]. The speed is faster. Tangle is also the first decentralized ledger system that does not require a fee. By distributing the work of verifying transactions to each trader, Tangle has saved the commission, which in turn increases work efficiency. Compared with other platforms, IOTA has a shorter time and there are still some imperfections waiting for the development team to improve [28].

With the development of blockchain technology, more and more blockchain platforms have come out one after another, and each platform has its own special emphasis on service projects. According to different application scenarios, appropriate development platforms should be selected, which are tough in the IoT. In the body update test, more attention is paid to stability, scalability, execution efficiency, and construction cost [29]. The stability determines the availability of the system. A system with high stability not only ensures the correct operation of the system, but also gives the user a good experience. The highly scalable system can effectively face various emergencies. When faced with an unknown attack method, it can have greater flexibility to deal with it. At the system development level, it can also expand its included fields by expanding new functions. Execution efficiency is one of the important comparison projects for any system. Efficiency and safety are roughly inversely related. It is always the goal of researchers to increase execution efficiency without reducing security [30]. For the IoT environment, there are tens of thousands of users. The cost of construction affects the user’s willingness to use the system, and the cost of construction can also effectively use the resources on the development system [31]. In addition to efficiency considerations, the strength of the system also includes the ability to resist attacks. The system with high system security has the ability to maintain the service. The above-mentioned features can be compared among different blockchain platforms, and we find Ethereum is superior to others. Therefore, we will use Ethereum as the development platform in this study.

## 3. Proposed Architecture and Mechanism

This section first introduces the security goals that must be considered for a secure firmware updating mechanism and then illustrates the architecture and mechanism of the proposed firmware update platform based on blockchain in detail.

### 3.1. Security Goals

The security goals that must be considered for a secure firmware updating mechanism are listed as follows:

(1) Firmware integrity: A secure firmware updating platform must ensure the integrity of uploaded firmware update files.
(2) Malicious code resistance: A secure firmware update platform must be able to help IoT devices resist a variety of malicious codes because IoT devices are lightweight and lack a strong malicious code scanning mechanism.
(3) Distributed denial-of-service (DDoS) resistance: When massive IoT devices request a firmware update at the same time, these requests will precipitate a DDoS problem. A secure firmware updating platform must be able to alleviate such a DDoS problem.

### 3.2. System Architecture

**Figure 1:**
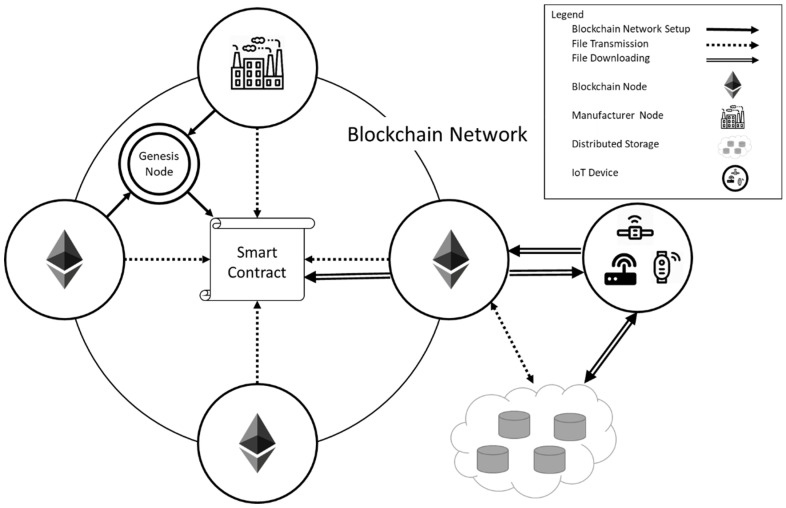
System architecture of the proposed firmware update platform.

There are three parts in the system, which contains “Blockchain Network Setup”, “File Transmission”, and “File Downloading”. The three parts will be introduced in detail in Section 3.3. The proposed system architecture has six roles, as shown in Figure 1.

There are six roles in the proposed system, including genesis node, smart contract, blockchain node, manufacturer node, distributed data storage, and IoT device. They will be explained as follows:

(1) Genesis node: The genesis node is responsible for defining the functions of the smart contract. After the completion of the smart contract, the genesis node exits the system or becomes a blockchain node, losing the ability of writing a smart contract.
(2) Smart contract: The smart contract contains functions that will be used in the system, including determining which node is an uploader and whether the firmware is authenticated and can be called by other blockchain nodes.
(3) Blockchain node: The blockchain node is responsible for verifying and uploading files, and in order to complete the assigned tasks, the blockchain nodes must be devices with high computing power, such as personal computers and company servers.
(4) Manufacturer node: The manufacturer node is one of the blockchain nodes. The difference between the manufacturer node and the blockchain node is that the manufacturer node has the function of uploading files, and the rest of the permissions are the same as the blockchain nodes.
(5) Distributed data storage: In order to reduce the burden on each node and improve the security of the system, the files are stored in a decentralized database, and only the necessary file information is stored in each node.
(6) IoT device: Because the IoT device is not one of the blockchain nodes, the device does not need to have powerful computing power and only needs to be able to communicate with the blockchain node. This method can increase the diversity of the system.

**Figure 2:**
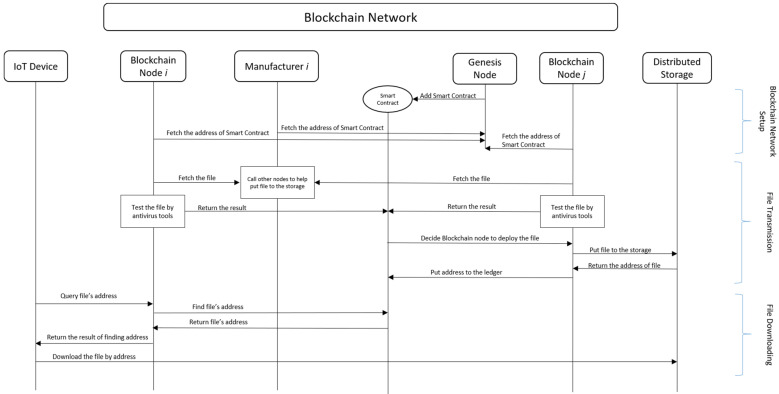
Process flow of the proposed firmware update mechanism.

In addition, the process flow of the proposed firmware update mechanism is shown in Figure 2, including the phases of the blockchain network setup, file transmission, and file downloading. In Figure 2, each step will be explained as follows.

(1) Writing a smart contract in the blockchain system by genesis node.
(2) Blockchain node and manufacturer node fetch the smart contract address.
(3) Genesis node exits the system or turns to a general blockchain node.
(4) The manufacturer node initiates a transaction of file uploading.
(5) Obtaining files by other blockchain nodes through smart contracts.
(6) The node participating in the verification performs out-of-chain antivirus and returns the result to the smart contract.
(7) The smart contract assigns one of the verification nodes to upload the file or reject the transaction of file uploading according to the verification result.
(8) The assigned verification node uploads the file to the distributed data storage and obtains the address.
(9) The assigned node records the file address into smart contract.
(10) The IoT device queries for a new firmware by blockchain node.
(11) The blockchain node queries through the smart contract whether there is an update file available for download.
(12) The blockchain node returns the query result to the IoT device.
(13) The IoT device downloads the update file or does not perform the action according to the result of the return.
(14) Steps (1) to (3) are “Blockchain Network Setup”, Steps (4) to (9) are “File Transmission”, and Steps (10) to (13) are “File Downloading”.

### 3.3. Proposed Mechanism

The system deploys smart contracts through the genesis node and obtains contract locations from other nodes. Then, the manufacturer node that wants to release the firmware update file initiates the transaction, and the file is transmitted to other nodes for verification. After the verification, the file is stored into the distributed database, and the file information is saved in the ledger for other nodes to query. The IoT device sends a query request through the blockchain node and downloads the update file according to the query result to complete the secure updating. The following subsections will describe the three phases of the proposed system, including the blockchain network setup, file transmission, and file downloading.

#### 3.3.1. Blockchain Network Setup

**Figure 3:**
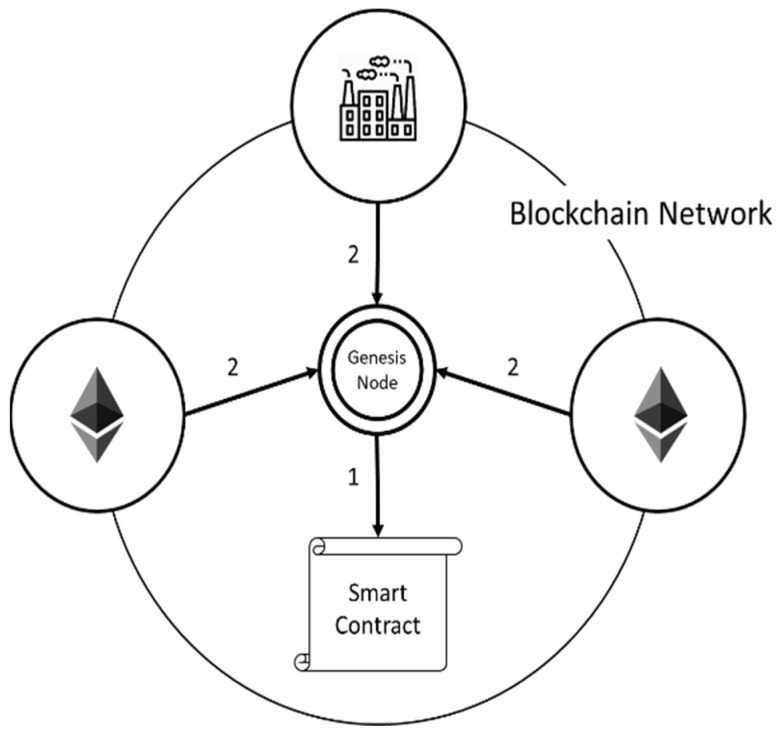
Blockchain network setup.

In the blockchain, a genesis node is required to deploy the first smart contract and tell the other nodes the contract location. After the deployment task has been completed, the node will leave the network or become a general node, as shown in Figure 3.

**Step** **1** The smart contract created by the genesis node, which defines the system functions, can increase the usability of smart contracts and avoid the inconvenience of writing smart contracts. When the blockchain network is setting up, the smart contract defines the function to avoid the writing of smart contracts conflicting with another node.
**Step** **2** The node that wrote the contract knows the address where the smart contract is stored. As a result, the node that wants to join the blockchain network, including the general blockchain node and the manufacturer node, needs to ask the genesis node for the contract address. After informing other nodes of the contract address, the genesis node exits the blockchain network or becomes a general node and thus loses the authority to write a smart contract.

#### 3.3.2. Firmware Update File Transmission

**Figure 4:**
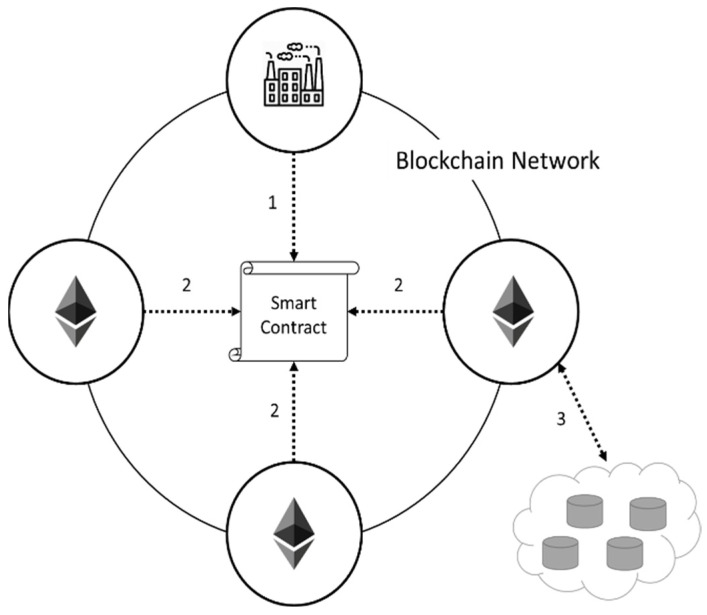
Firmware update file transmission.

In order to maintain the operation of the blockchain-based system, all nodes on the network will have the same record, which is called a ledger. If the firmware update file is directly stored on the ledger, it will waste resources and lower the convenience of use. To improve the blockchain-based system, we use a decentralized database to store the file, while the ledger only stores the address of the file. The node that wants to upload the file sends a request to the smart contract and waits for other nodes to process it, including checking whether the file is secure or not. After the processing, the smart contract specifies a node participating in processing. The node uploads the file to the decentralized database and then records the file address into the ledger to complete the file transfer, as shown in Figure 4. Each step will be listed in detail below, and the pseudocode of the major smart contract function in the system will be described here.

**Step** **1** The manufacturer who wants to upload the firmware update file sends the request through the smart contract and transmits the file to other blockchain nodes for verification. This method ensures that the manufacturer cannot change the file after being deployed. The manufacturer needs to call the upload file function before uploading the file and provide the update file address to complete the upload action. In the smart contract, the address of the manufacturer node is mapped to a column in the file information. It will initialize the parameters in the column, including firmware original address, download point, the number of nodes that execute firmware update validation but pass or fail the validation, whether to complete validation, and the address of the uploader. The file uploading is shown in Algorithm 1.

**Algorithm 1:** File uploading**Input:** manufacturer_address, file_address **Output:** True/False N ← mapping (manufacturer_address) Initialize File_information [ N ] //File_address ← file_address                //Download_point ← NULL                //Pass ← 0                //Reject ← 0                //Check ← false                //Finish ← false                //Uploader ← NULL

In Algorithm 1, the manufacturer needs to call the uploading function before uploading the file and provide the update file address (*file_address*) to complete the upload action. The manufacturer node address (*manufacturer_address*) will map to a column (*N*) of file information. Then, the parameters in file information will be initialized, including original location of the firmware (*File_address*), the address of validated firmware (*Download_point*), the number of nodes that complete firmware update validation but pass the validation (*Pass*), the number of nodes that complete firmware update validation but fail the validation (*Reject*), whether to complete the validation (*Check*), whether to complete the uploading (*Finish*), and the address of uploader (*Uploader*).

**Step** **2** When the manufacturer sends the request, the other nodes can verify the file provided by the manufacturer. The verification method needs to execute on a local host, which the verification is off-chain processing in each node. The reason that we do not verify the file on the blockchain is to enhance the varieties of virus code detection. By comparing the virus code, off-chain processing is more reliable than the inspection by the specific file check node. The virus scanning for firmware files is shown in Algorithm 2.

**Algorithm 2:** Virus scanning for firmware files**Input:** manufacturer_address **Output:** True/False N ← mapping (manufacturer_address) Get File_address by File_information [ N ] Download file F by File_address **if** F passes antivirus tool, **then**    Pass ← Pass + 1  //in File_information [ N ] **else**    Reject ← Reject + 1 //in File_information [ N ] **end if**

In Algorithm 2, nodes obtain the file (F) from the original file address in the file information and log the result of the file validation to the contract (Pass + 1 or Reject + 1). The nodes in the proposed system have the same permissions, so there is no node that can influence the result by itself.

**Step** **3** The verified firmware update file is transmitted to the distributed file server by the blockchain node, and the distributed file server returns the address where the file is stored. Finally, the address returned by the distributed file server is stored in the ledger. If the file does not pass the verification, the node participating in the verification notifies the manufacturer that the file upload request is not successful. The biggest weakness of blockchain is that the requirement of storage space is too large. By storing files in the distributed file server, only the file addresses are stored on the ledger, and therefore they can reduce the needs of system storage. Additionally, the distributed file server is stronger to defend DoS attacks than a single file server, so this proposed mechanism has high system security. In this step, the smart contract decides whether to upload based on the result of each nodes’ returns. If the file is verified, the smart contract will assign a node participating in the verification to upload the file to the distributed data storage. At the same time, the record information is recorded in the file information. In the following, the procedure of the uploader assigning is first shown in Algorithm 3, and the uploading of file download point is shown in Algorithm 4.

**Algorithm 3:** Uploader assigning**Input:** manufacturer_address **Output:** True/False N ← mapping (manufacturer_address) Get Pass by File_information [ N ] Get Reject by File_information [ N ] **if** Pass + Reject > threshold_1 **and**    Pass/(Pass + Reject) > threshold_2 **then**              //threshold_1 and threshold_2 are defined by genesis node    Uploader ← one of validation nodes’ addresses             //Uploader is in File_information [ N ] **else**    Uploader ← NULL    Check ← True //Check is in File_information [ N ] **end if**

**Algorithm 4:** Uploading of file download point**Input:** manufacturer_address, node_address **Output:** True/False N ← mapping (manufacturer_address) Get Uploader by File_information [ N ] **if** Uploader == node_address **then**   Download_point ← download_point             //“Download_point” is in File_information [ N ]             //“download_point” is file address in distributed database   Finish ← True //in File_information [ N ] **else**   print(“Cannot Update File Information”) **end if**

In Algorithm 3, the smart contract decides whether to upload firmware according to the result of each node’s return. In order to maintain the correctness of the system, it must be ensured that there are enough nodes to participate in the validation and the passing rate. As a result, we need to decide two thresholds to ensure the number of nodes (*threshold_1*) and passing rate (*threshold_2*). If the firmware passes the validation, the smart contract will assign a validating node to upload the firmware to distributed data storage. At the same time, the assigned node records the file address (*Download_point*) to the file information in Algorithm 4.

#### 3.3.3. Firmware Update File Downloading

**Figure 5:**
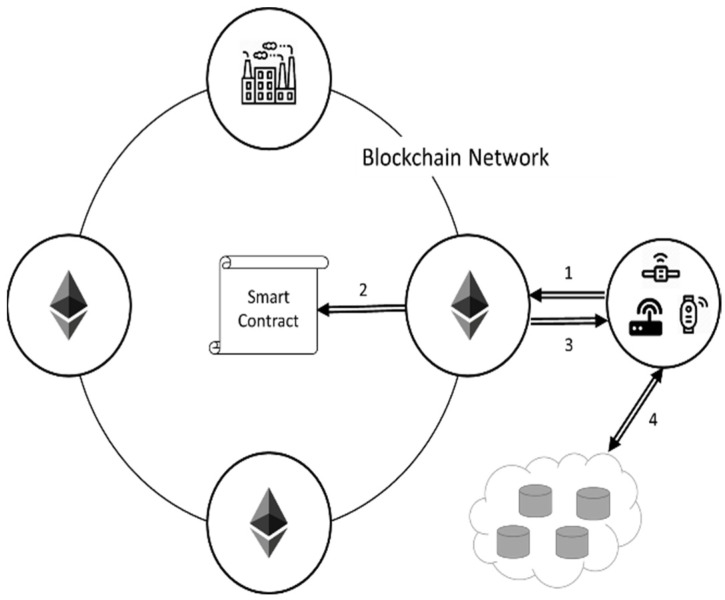
Firmware update file downloading.

Only nodes on the blockchain network can access the smart contract. The IoT device that wants to download firmware needs to send a request to a blockchain node, then the blockchain node finds out whether a suitable firmware is available for downloading, and it finally sends the response to the IoT device. The IoT device downloads the file from the distributed database according to the received response and completes the update action, as shown in Figure 5. In order to strengthen the security of the interaction between the IoT device and the blockchain node, the following steps will be executed before the IoT device connects to the blockchain.

- Hardware security module (HSM): In the proposed platform, we adopt a tamper-proof hardware security module [32,33] in the IoT device to store the important system parameters securely.
- Key generation procedure: The HSM is responsible for generating the pseudo-identity and corresponding public/private keys for its own IoT device.

In Figure 5, the four steps for firmware update file downloading are depicted as follows.

**Step** **1** The IoT device that wants to execute the firmware update sends a request to a blockchain node and transmits the corresponding device type and current version so that the blockchain node can confirm whether an update file is available for download. Since the blockchain node needs to have the computing power for verification, and the IoT device does not necessarily have sufficient computing power, it cannot become a member of the blockchain nodes.
**Step** **2** After receiving the IoT device request, the node checks whether there is an eligible update file by the smart contract. As the data on the chain will increase over time, the system has to find the data on the chain quickly. Through the smart contract, it is more efficient than the local search and can determine the correctness of the results.
**Step** **3** The blockchain node informs the IoT device of the result of the request. If the information of the updated file is recorded on the ledger, the result of the return is the downloaded address of the file, and the IoT device can download the file through the address. If the information of the update file cannot be found, blockchain node returns the message “No update file is available for downloading.”
**Step** **4** The IoT device downloads the file by using the file address obtained from the blockchain node. The correctness of the file downloaded through the above steps can be ensured by the blockchain features and consensus mechanism.

## 4. Security Analysis

SUIT (Software Updates for Internet of Things) is a new IETF (Internet Engineering Task Force) standard for secure IoT firmware updates, and typical threats against a firmware update solution are discussed in the SUIT information model [34]. Therefore, we assess the security of the proposed blockchain-based firmware update scheme based on these threats, focusing on the security goals of firmware integrity, malicious code resistance, and DDoS mitigation. In addition, suggestions for secure smart contracts and security comparisons among several related schemes are also discussed in this section.

### 4.1. Secure IoT Firmware Update System

**Property** **1.** *With the characteristic of irreversibility in the blockchain system, the integrity of uploaded firmware update files can be greatly strengthened.*

When manufacturers require updating their firmware, they must prepare the firmware checksum. Then, a blockchain node executes the smart contract to confirm the correctness of the checksum with the help of off-chain programs. The firmware updating transaction can be finished if, and only if, the checksum is valid. At last, the checksum will be recorded in the blockchain. Based on the blockchain analysis [35], the probability of changing information in a blockchain can be analyzed as follows. Assume that *a_z_* is the probability that an attacker will be able to catch up when he/she is currently *z* blocks behind. Besides, assume that *n* is the number of blocks found by the honest network with probability *p* in average time, and that *m* is the number of blocks found by the attacker in that time, where *m* is more accurately a negative binomial variable. Therefore, it is the number of successes (blocks found by the attacker) before *n* failures (blocks found by the honest network), with a probability *q* of success. Based on Rosenfeld’s analysis result [35], it follows that the probability of changing to succeed, when someone waits for *n* confirmations, is equal to:

(1) $$\begin{array}{l} \overset{\infty }{\underset{m=0}{\sum }}P\left(m\right)a_{n-m-1}\\ =\overset{n-1}{\underset{m=0}{\sum }}\left(\begin{array}{c} m+n-1\\ m \end{array}\right)p^{n}q^{m}{\left(\min\left(q/p,1\right)\right)}^{n-m}+\overset{\infty }{\underset{m=n}{\sum }}\left(\begin{array}{c} m+n-1\\ m \end{array}\right)p^{n}q^{m}\\ =\{\begin{array}{ll} 1-\overset{n}{\underset{m=0}{\sum }}\left(\begin{array}{c} m+n-1\\ m \end{array}\right)p^{n}q^{m}-p^{m}q^{n} & \mathrm{if}\;q<p\\ 1 & \mathrm{if}\;q\geq p \end{array} \end{array}$$

According to Rosenfeld’s analysis result from Equation (1) [35], the changing success rate of two confirmations is less than 10%, four confirmations is less than 1%, and six confirmations is less than 0.1%. As a result, the security level of the checksum increases in strength as long as the growth of blocks continues.

On the other hand, because “Step 1” in Section 3.3.2 is executed, the manufacturer needs to release the firmware file when uploading it, and then the file is uploaded to distributed storage by a validating node. Afterwards, a device will download the file from the distributed database, so the manufacturer cannot modify the updated file. This feature prevents the device from downloading the firmware that is tampered from attacking the manufacturer’s server.

**Property** **2.** *Based on the validation result of multi-node antivirus, the proposed scheme can prevent IoT devices from downloading malicious updated firmware.*

In 2019, a hacker was found to install the backdoor malware on the user’s computer through Asus’s official server [36]. The hacker used a legal certificate to make it look like a legitimate update at the time of installation and obtains device information to launch an attack. This incident is an attack launched against the manufacturer’s server so that the device is implanted with malicious programs.

According to “Step 2” of Section 3.3.2 in the proposed mechanism, multi-node antivirus is performed before the firmware file is uploaded to the distributed database, ensuring that the stored file is not implanted with malicious code. The multi-node voting method determines the file can be uploaded if it has passed the validation of more than half of the validation nodes. The threshold of validation passing ratio to determine whether malicious code is present can be adjustable. The attacker needs to control more than half of the validation nodes to affect the validation result, which is too hard to succeed. Thus, the proposed firmware update scheme can effectively accomplish malicious code resistance.

**Property** **3.** *Based on decentralized blockchain system and smart contract execution, the DDoS problem can be alleviated.*

When massive IoT devices request a firmware update at the same time, these requests will precipitate a DDoS problem. In the proposed scheme, the smart contract for processing update requests is performed in a distributed way. Additionally, the firmware files are also stored on a distributed peer-to-peer file sharing system. Therefore, such a DDoS problem can be alleviated by the proposed decentralized blockchain system and smart contract execution.

On the other hand, Satori botnet appeared in 2018, hijacking countless IoT devices and infecting many routers with different IP addresses. It used the malicious software Brickerbot to launch a PDoS (Permanent Denial of Service) attack on the device, which attacked the IoT device through SSH (Secure Shell) Crawler, Telnet Crawler, etc., and performed a malicious firmware update on the device [37]. According to “Step 1” of Section 3.3.3 in the proposed mechanism, IoT devices are to connect to blockchain nodes. By connecting to a more reliable blockchain node and avoiding direct connection to a centralized server for updating a firmware file, the connection process of IoT devices can have a higher security protection and reduce the probability of IoT devices suffering from the PDoS attack.

### 4.2. Suggestions for a Secure Smart Contract

In the following, we will analyze and explain the smart contract’s security [38], including some serious vulnerabilities, “The DAO Attack” and “GovernMental Attack”, and give further suggestions for secure smart contracts.

#### 4.2.1. The DAO Attack

The DAO (Decentralized Autonomous Organization, DAO) attack [39] used the vulnerabilities of the smart contract to make repeated withdrawals, and the vulnerability exploits the characteristics of the fallback function in the smart contract. By interrupting the execution of the smart contract, the purpose of re-depositing before the deduction is achieved. This incident is a major attack of Ethereum, which is solved finally by using a hard fork to restore the blockchain to the pre-attack state, while letting users pay attention to such recursive attacks.

This type of recursive attack exploits the characteristics of a function declaration in a smart contract, allowing an attacker to launch an attack using a smart contract with an attacking nature. As a result, the way to prevent such attacks is to limit the use of contracts not to be other smart contracts. In this study, based on the characteristics of the traceable source in the blockchain, we add the “initial source of the query message” to the smart contract and check whether the “original source of the message” and the “message source” are the same. Therefore, we can prevent such attacks by verifying whether the contract user has passed through a self-written contract to initiate a transaction.

#### 4.2.2. GovernMental Attack

Ethereum can develop many applications in addition to cryptocurrency transactions. By writing smart contracts, we can use Ethereum to play games on the platform of Ethereum. There is a game called “GovernMental”, in which participants can continuously send Ethercoin to the contract. Moreover, when the contract does not receive any transaction within 12 h, all the Ethereum in the contract can be taken by the “participant who sent the last transaction to the contract”. However, the author of the game needs a lot of gas features to reproduce the contract status, so that all participants cannot take the Ethereum in the contract, which is one of the famous Ponzi schemes in Ethereum [40]. An attacker used the loophole of the contract to repeatedly consume the gas in the contract so that the contract cannot successfully complete the transaction again after a small number of transactions, thereby making the “re-contract status” require less than the “completed transaction”. The gas required at the time allows the game to end normally, undermining the author’s original purpose.

This event reminds us that the use of gas should be very careful when writing a smart contract. When using the contract function, it can check the amount of remaining gas before checking the transaction to avoid the unexpected situation caused by insufficient gas.

### 4.3. Security Comparisons among Several Related Schemes

Lee and Lee [11] proposed a blockchain-based firmware verification and update method. Their mechanism stores firmware files into nodes in the blockchain, and IoT devices also belong to one of the blockchain nodes, making all IoT devices need to store all the firmware files in the chain. This design makes firmware files not easy to be tampered with, but it is difficult to implement in reality due to resource limitations on IoT devices, because the devices are directly connected to the Internet. It is easy to expose device-related information to attackers. In addition, because the IoT device does not necessarily have high-intensity computing power, it is prone to delay when the node synchronizes. In such a way, an attacker can launch related attacks through the delay of synchronization, thereby causing the system to be harmed.

Boudguiga et al. [12] also proposed a blockchain-based method for updating the firmware of IoT devices by including different vendors and antivirus companies in the blockchain system and requesting the firmware files in the system. It must be verified by the antivirus company node before it can be synchronized by other blockchain nodes in the system, and then IoT devices request the files from the blockchain node. This design is submitted to the antivirus company for the verification work. The number of verification nodes is very rare, and thus the verification node has a large burden. And the attacker only needs to attack a few nodes to enable harm to the system.

Yohan and Lo [13] also proposed a blockchain-based network firmware update method that requires manufacturers to upload firmware files to supervise each other. Moreover, in order to reduce the burden on nodes, the URI (Uniform Resource Identifier) of the file is synchronized in the node. After verification, the URI is broadcast to each node. Then, the URI is obtained by IoT devices, and the updated file is downloaded to the corresponding vendor database to complete the firmware update. In this method, the synchronization is via URI, and the file is managed by each vendor. Therefore, even if the verification is performed at the time of synchronization, there is no guarantee that the firmware file is still the same after a certain period of time, and attacks on vendors’ databases can cause harm to the system.

In contrast, all the nodes in this study have the authority to verify firmware files updates, and this is performed through multiple antivirus inspections to ensure the file is safe. Besides, after the verification is completed, the proposed mechanism uploads the files to the decentralized database so that the firmware manufacturer cannot change the file again. That is, regardless of whether the manufacturer is attacked, the files verified by the proposed system cannot be changed, and the distributed database can also be used to effectively protect the security of the files [41]. Furthermore, in this study, the blockchain node is commissioned by devices for querying whether the system has a new version of firmware update file, so that the devices can be protected from being exposed to a dangerous environment.

## 5. Experimental Evaluation and Comparison

This section first depicts the experimental environment based on the Ethereum blockchain and distributed storage system Swarm and then presents two evaluation procedures for measuring the performance of firmware uploading and downloading. In addition, functionality comparisons among several related schemes are also discussed in this section.

### 5.1. Experimental Environment

**Figure 6:**
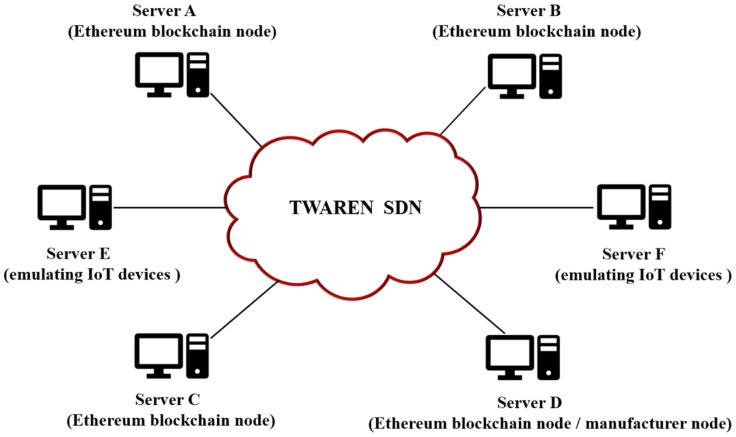
The network topology of experiment for the proposed system.

The experimental evaluation environment is shown in Figure 6. In the experimental environment, we have constructed the system with the four servers, A, B, C, and D, selected to be the Ethereum blockchain nodes in a private network over the TWAREN (TaiWan Advanced Research and Education Network) SDN (Software Defined Network) environment. Servers A, B, C, and D are deployed in different physical locations, and each of them is equipped with 16-cores Intel Xeon E5630 @ 2.53 GHz and 16 GB RAM, running the Ubuntu 18.04 LTS. Server D is simulated as a manufacturer node that has different sizes of firmware update files ranging from 31.8K to 5.6M. To emulate the IoT devices environment, we built a Raspberry Pi QEMU image running on servers E and F equipped with 16-cores Intel Xeon E5620 @ 2.4 GHz and 72 GB RAM. We enable the docker service in QEMU for the benchmark setup. A dockerized version of ApacheBench benchmarking tool from “https://hub.docker.com/r/adamoss/rpi-apachebench/” (accessed on 15 October 2021) is used to measure the performance of the proposed system, where ApacheBench is a tool for benchmarking an Apache HTTP (HyperText Transfer Protocol) server, showing how many requests per second the Apache installation is capable of serving. Although ApacheBench is originally designed to test the Apache HTTP server, it is generic enough to test any web server. Moreover, we use Swarm as our firmware storage system, where Swarm package is a distributed storage protocol and content distribution service in Ethereum. And the smart contracts used in the proposed mechanism are developed by Solidity program language.

### 5.2. Evaluation of Firmware Uploading

The following will explain and compare the experimental results with other methods in terms of firmware uploading. In this evaluation, we compare the firmware uploading and evaluate the system efficiency by the number of uploaded firmware files received at the same time. Since Yohan and Lo [13] have compared the efficiency of their scheme with Lee and Lee’s [11] and Boudguiga et al.’s schemes [12] and confirmed that it is superior to the other two in terms of efficiency, we will focus on comparing with Yohan and Lo’s scheme [13].

When conducting an experiment, the smart contract can display the status of the current transaction, including the block number of the transaction, the generation time of the block, and the gas spent on the transaction. In the process of setting the blockchain system, the time required to generate each block can be adjusted, and the number of received file upload requests can also be set through smart contracts. Table 1 shows the experimental result of the proposed firmware uploading with different quantities of firmware files uploaded.

**Figure 7:**
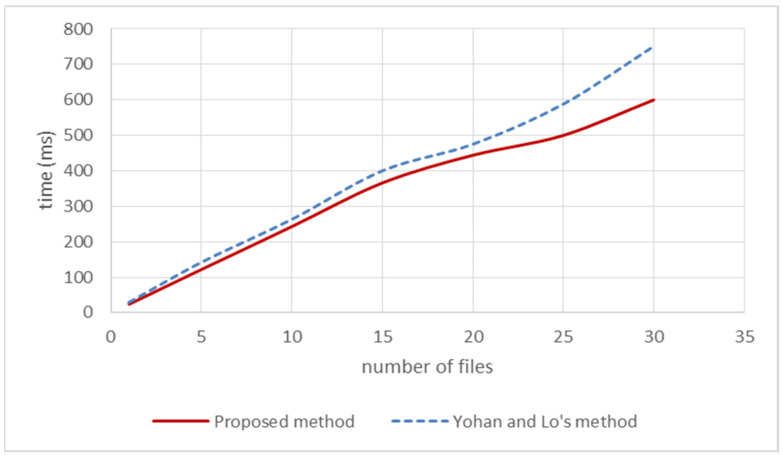
Comparison of the time cost in firmware uploading.

**Figure 8:**
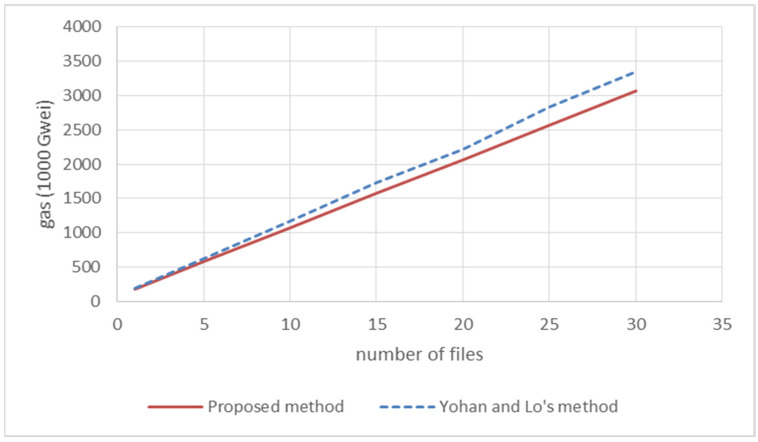
Comparison of the gas cost in firmware uploading.

The items listed in Table 1 include gas cost, timestamp, block number, and the number of firmware files. “Gas cost” represents the total price of the experiment, where the price unit is Gwei and 1 Gwei is equal to $10^{-9}$ Eth. When the number of uploaded firmware files is 30, the total price of firmware uploading is equal to 3,061,073 × 10^−9^ Eth. “Timestamp” represents the timestamp of the block generation, from which the time for generating each block can be inferred. By combining the data of each field, we can know the execution time and gas cost, as shown in Figure 7 and Figure 8, where the blue dotted line represents the method of Yohan and Lo [12], and the red solid line represents the proposed method.

In Figure 7 and Figure 8, we can see that the proposed method has better performance when receiving multiple uploading requests at the same time. The processing time can reach 20 milliseconds when receiving a single uploading request, and the proposed system requires less gas cost in computing. Combined with the security analysis in Section 4.1 and the efficiency comparison in this subsection, it can be found that the proposed system has enhanced the security and the runtime efficiency for firmware uploading.

### 5.3. Evaluation of Firmware Downloading

**Figure 9:**
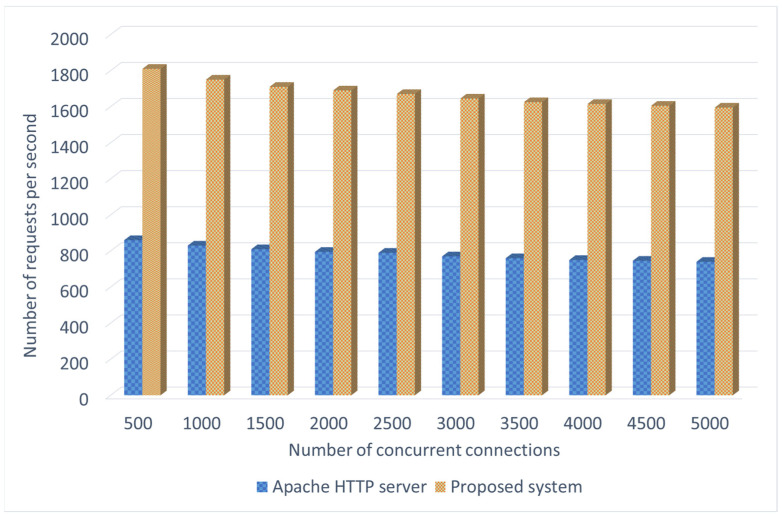
Comparison of the performance of firmware downloading.

In the experiment of firmware downloading, we measure the number of requests per second that the proposed system can handle when concurrent IoT devices execute the downloading procedure. As long as IoT devices sends the firmware updating query to the blockchain node, the smart contract will handle this request. It checks whether the system has a new version of firmware and then sends the downloading link to the IoT device. In this evaluation, the ApacheBench benchmarking tool is used to simulate the requests sent from IoT devices. We compare the proposed system with an Apache HTTP (HyperText Transfer Protocol) server based on downloading the firmware update file whose size is 31.8 K. For the 31.8 K update file, the proposed system can support more than 1500 requests per second even if the number of concurrent connections gradually increases to 5000. As shown in Figure 9, it can be found that our proposed system has much better performance than the Apache HTTP server. Furthermore, the problem of single point of failure does not occur in the proposed system.

### 5.4. Functionality Comparisons

This paper proposes a blockchain-based IoT security update system with a distributed database, which improves the inadequacies of the existing methods. The proposed approach is based on the technology of blockchain to ensure that IoT devices obtain their genuine firmware. Moreover, the proposed approach can reduce the needs of node storage due to the use of a distributed database. In the following, we will list the advantages and disadvantages of the methods mentioned above in terms of the items required for security analysis and comparison based on the literature [42,43,44], as shown in Table 2.

From Table 2, we can see that the proposed method uses a distributed database to reduce the total space requirement of the system. Besides, because the files are not managed by manufacturers, it can effectively prevent the manufacturers from tempering the files after the file stored in the database. The system allows multiple manufacturers to operate simultaneously, so the proposed method is suitable for heterogeneous IoT device networks.

## 6. Conclusions

With the increasing number of IoT devices, more sophisticated security mechanisms are needed. This study is based on the blockchain technology to achieve multi-node firmware verification, and therefore IoT device security can be accomplished. The contributions of this study are listed as follows:

(1) This research proposes a method of using a distributed database to reduce the storage space by storing firmware information on a ledger, instead of storing firmware itself.
(2) After downloading the firmware in the proposed system, the correctness and integrity of the obtained firmware of IoT devices can be ensured.
(3) IoT devices download the firmware through the download point of the distributed database, instead of going through the manufacturer.
(4) This study does not only reduce the burden of manufacturers’ servers, but also prevents the manufacturers from tampering the firmware after deploying it.

However, our mechanism does not implement the revocation function of expired IoT firmware in the decentralized storage system. This is our research direction in the future.

## Figures and Tables

**Table 1:** The experimental result of the proposed firmware uploading.

Gas Cost (Gwei)	Timestamp (s)	Block Number	Files
188,941	1,559,662,521	110	1
585,094	1,559,662,521	110	5
1,080,286	1,559,662,521	110	10
1,575,481	1,559,662,521	110	15
2,070,677	1,559,662,522	111	20
2,565,874	1,559,662,522	111	25
3,061,073	1,559,662,523	112	30

**Table 2:** Functionality comparisons.

	Method	Proposed Method	Lee and Lee’s Framework [11]	Boudguiga et al.’s Framework [12]	Yohan and Lo’s Framework [13]
Feature					
Firmware integrity		Yes	Yes	Yes	No
Non-disclosure of IoT device position		Yes	No	Yes	Yes
Satori botnet defending		Yes	No	Yes	Yes
No impact of manufacturer server being attacked		Yes	No	No	No
Nodes of verification participation		All	Partial	Partial	Partial
Device anonymity		Yes	No	Yes	Yes

## Data Availability

The data used to support the findings of this study are available on request from the corresponding author.
